# The role of probiotics in vaginal health

**DOI:** 10.3389/fcimb.2022.963868

**Published:** 2022-07-28

**Authors:** Zhaojun Mei, Dandan Li

**Affiliations:** ^1^ Luzhou Maternal and Child Health Hospital, Luzhou Second People’s Hospital, Luzhou, China; ^2^ University of Chinese Academy of Sciences, Beijing, China

**Keywords:** probiotic, vaginal health, human papilloma virus, *lactobacillus*, vaginal microenvironment

## Abstract

Probiotics have been widely used in the treatment of intestinal diseases, but the effect of probiotics on female reproductive tract health is still controversial. *Lactobacillus* is the most abundant microorganism in the vagina, which is related to the vaginal mucosal barrier. *Lactobacillus* adheres to the vaginal epithelium and can competitively antagonize the colonization of pathogens. The factors produced by *Lactobacillus*, such as bacteriocin and hydrogen peroxide (H_2_O_2_), can inhibit the growth of pathogenic microorganisms and maintain the low pH environment of the vagina. Probiotics play an important role in maintaining the stability of vaginal microenvironment, improving immune defense and blocking the progression of cervical cancer. We review the research progress of probiotics represented by *Lactobacillus* in gynecological diseases such as human papilloma virus (HPV) infection, bacterial vaginosis (BV) and Genitourinary Syndrome of Menopause (GSM), so as to provide basis for further exerting the role of probiotics in women’s health.

## Introduction

Vaginal microenvironment is composed of normal flora in vagina, endocrine regulation and mucosal epithelial barrier (Saraf et al., 2021). There are many microbial colonization in the vagina of healthy women, among which *Lactobacillus* plays a major role (95%) (Ilhan et al., 2019). Based on the different species of specific *Lactobacillus*, it can be divided into five different community state types (CSTs). Among them, CST I, II, III and V are mainly *L. crispatus*, *L. gasseri*, *L. iners* and *L. jensenii* respectively, while CST IV is on the contrary (Borgogna et al., 2020; Langner et al., 2021). It is represented by the reduction of lactic acid bacteria and there are strictly anaerobic species, such as *Gardnerella*, *Megasphera* and *Prevotella.* Among them, *L. crispatus*, *L. gasseri* and *L. jensenii* can produce lactic acid and H_2_O_2_, acidify the vaginal environment to pH < 4.5, and inhibit the growth of other viruses and bacteria (Anderson et al., 2014; Das et al., 2022). However, *L. iners* is considered to be a *Lactobacillus* in an excessive state (Pramanick et al., 2019). In addition, the metabolites produced by *Lactobacillus* can also stimulate the host to produce antimicrobial peptides and anti-inflammatory cytokines (Niu et al., 2017). Vaginal epithelial cells change periodically under the action of estrogen and progesterone. Glycogen produced in this process provides energy for the growth of *Lactobacillus*. *Lactobacillus* can also prevent invasive pathogens from adhering to vaginal epithelium through competitive rejection (Han and Ren, 2021).

Vaginal microecological balance is a dynamic process. Slight vaginal flora imbalance can be regulated by itself. Serious flora imbalance will lead to gynecological diseases (Chao et al., 2019; Zhang et al., 2021). It is well known that cervical cancer is a cancer associated with HPV. Many studies have proved that the composition of vaginal microorganisms is related to the development of high-risk HPV infection and cervical lesions (Mitra et al., 2015; Jang et al., 2017; Mitra et al., 2020). The abundance of BV related bacteria, such as *Gardnerella*, may also increase the risk of cervical lesions during HPV infection (Wei et al., 2020). When women enter menopause, due to the decrease of estrogen level, the content of glycogen in epithelial cells and the number of *Lactobacillus*, pathogenic bacteria are easy to invade and reproduce, resulting in senile vaginitis (Athanasiou et al., 2016). Probiotics are a kind of active microorganisms that colonize the human intestinal tract and reproductive tract and are beneficial to the host. A large number of studies have proved that oral probiotics can treat a variety of digestive system diseases. *Lactobacillus*, as the most dominant bacterial species in the vaginal microenvironment, can maintain or change the vaginal microecological balance (Łaniewski et al., 2020; Piccioni et al., 2021). In this review, we focus on the role of probiotics in maintaining vaginal health in women.

## Probiotics and cervical cancer

Cervical cancer is the first malignant tumor of female reproductive tract, and about 300000 people die of cervical cancer every year (Jahanshahi et al., 2020; Kovachev, 2020). When infected with HPV, it can destroy the vaginal microecological balance, reduce the number of *Lactobacillus* and increase the adhesion and colonization of abnormal flora. This further leads to the up regulation of HPV protein expression, promotes the development of cervical intraepithelial neoplasia (CIN), and even leads to the occurrence of cervical cancer (Curty et al., 2019). Gao et al. (Gao et al., 2013) were the first to systematically evaluate the relationship between vaginal microbiota and HPV infection and found that vaginal bacterial diversity in HPV-positive women was more complex and the composition of vaginal microbiota was different. A study (DI Pierro et al., 2021) demonstrated for the first time that oral *Lactobacillus curlicus* can change the state of CST and increase HPV clearance. Persistent high-risk HPV infection and changes in cervical microenvironment promote the development of cervical precancerous lesions (Liu et al., 2020). *Lactobacillus* activates the immune system to inhibit the proliferation of malignant tumors by secreting various antitumor metabolites, including phosphorylated polysaccharides and extracellular polysaccharides. (Champer et al., 2018; Pourmollaei et al., 2020). There is an important link between increasing probiotic intake and reducing cancer progression.

### Probiotics act directly on cervical cancer cells

As a kind of vaginal probiotics, *Lactobacillus* can not only acidify the vaginal environment, stabilize the vaginal flora and enhance the function of vaginal epithelial cells, but also kill cervical cancer cells. *Lactobacilli* adsorb and occupy the vaginal epithelium, preventing the adhesion of aggressive pathogenic bacteria that cause malignancies (Abdolalipour et al., 2020). *Lactobacillus* can inhibit cancer cell proliferation by secreting peptidoglycan and exopolysaccharides. Probiotics mainly enhance the immune process of the body, promote the production of cytokines, and inhibit the proliferation of monocytes. Recent studies have shown that probiotics such as *Lactobacillus casei* and *Lactobacillus rhamnosus* play an anticancer role by activating the maturation of NK cells and dendritic cells (Li et al., 2020; Kandati et al., 2022). *Lactobacillus* can also affect cellular and humoral immunity, promote the proliferation and differentiation of thymus derived cells, and further promote the immune recognition and proliferation of bone marrow-derived cells (Medina-Contreras et al., 2020). In addition, probiotic metabolites also have cytotoxic effects on cervical cancer cells. Wang et al. (Wang et al., 2019) found that the increase of *Lactobacillus* spp. was related to the decrease of the detection rate of high-risk subtype HPV infection, cervical intraepithelial neoplasia and cancer. Microbiota plays an increasingly important role in cancer and treatment (Xie et al., 2020). As a promising non chemotherapy alternative therapy, *Lactobacillus* has attracted extensive attention in restoring and maintaining normal vaginal flora and treating cervical cancer. As shown in **Table 1**, the effects of probiotics on cervical cancer cells are summarized.

**Table 1:** Experimental studies of probiotics in cervical cancer.

Probiotics	Sources	Cell line	Results	Ref.
*Lactobacillus plantarum* 5BL	Vaginal secretions	HeLa	*Lactobacillus* can inhibit the activity of HeLa cells.	Nami et al., 2014
*Lactobacillus gasseri* and *Lactobacillus crispatus*	Commercial probiotics	HeLa	Supernatant of *Lactobacillus* is cytotoxic to cervical cancer cells.	Motevaseli et al., 2013
*Lactobacillus gasseri*	Vaginal secretions	HeLa	*L. gasseri* inhibits Hela cell proliferation and shows anti-inflammatory by reducing TNF-α.	Sungur et al., 2017
*Bifidobacterium adolescentis* SPM1005-A	Fecal samples	SiHa	*Bifidobacterium* can inhibit the expression of E6 and E7 oncogenes in SiHa cells.	Cha et al., 2012
*Lactobacillus rhamnosus* and *Lactobacillus crispatus*	Commercial probiotics	HeLa	Supernatants of these two *Lactobacilli* were cytotoxic to HeLa cells.	Nouri et al., 2016
*Lactobacillus crispatus, L. jensenii*, and *L. gasseri*	Commercial probiotics	Caski	Supernatant of *Lactobacillus* inhibits cervical cancer cells by regulating HPV oncogenes and cell cycle related genes.	Wang et al., 2018
*Lactobacillus casei* and *Lactobacillus paracasei*	Human breast milk	HeLa	Supernatant of *Lactobacillus* inhibits HeLa cells by regulating the expression of apoptotic genes.	Riaz Rajoka et al., 2018
*Lactobacillus* DM8909	Laboratory culture	HeLa and U14	*Lactobacillus* can inhibit HeLa and U14 cell migration by upregulating E-cadherin expression.	Li et al., 2017
*Lactobacillus crispatus* and *Lactobacillus rhamnosus*	Commercial probiotics	HeLa	Probiotic supernatant can inhibit the proliferation of HeLa cells by down regulating HPV oncogene.	Motevaseli et al., 2016

### Probiotics reduce the side effects of radiotherapy for cervical cancers

Radiotherapy is one of the main methods for the treatment of cervical cancer, but there are many side effects, the most common of which is radiotherapy-induced diarrhea (RID), which brings a greater burden to patients (Hombrink et al., 2000; Jahanshahi et al., 2020). Probiotics have shown good effects in the treatment of digestive system diseases and can alleviate the adverse reactions caused by inflammation. Probiotics can be added to reduce the side effects of radiotherapy for cervical cancer and enhance the antitumor effect. A study (Okawa et al., 1993) of 228 patients with stage IIIB cervical cancer showed that patients receiving probiotics as adjuvants had longer survival than patients receiving radiotherapy alone. In another meta-analysis (Qiu et al., 2019) comparing the incidence of probiotics in the prevention of diarrhea caused by cervical cancer radiation therapy, the probiotics group had a lower incidence of RID, RR 0.61 (95% CI 0.46-0.81; *P* = 0.0007). Negi et al. (Negi et al., 2020) developed cisplatin and probiotic bioburden pessaries for the treatment of cervical cancer. Histopathological studies showed that the preparation was safe for local administration of cisplatin. More research, especially clinical trials, is needed to understand the specific mechanisms by which probiotics can alleviate the side effects of radiation therapy for cervical cancer. As shown in **Table 2**, the research of probiotics in preventing or reducing the adverse effects of cervical cancer treatment on gastrointestinal tract is summarized.

**Table 2:** Studies with the role of probiotics in the prevention of RID therapy for cervical cancer.

Probiotics	Methods	Findings	Ref.
*Lactobacillus acidophilus* plus *bifidobacterium bifidum*	Patients who received cisplatin and pelvic radiotherapy were divided into a probiotic group and a placebo group.	Probiotics reduced the incidence of RID and improved stool consistency.	Chitapanarux et al., 2010
*Lactobacillus acidophilus* LA-5 plus *Bifidobacterium animalis* subsp	Patients were randomized to a probiotics group (containing 75 billion live freeze-dried bacteria) or a placebo group.	Probiotics reduced the incidence of diarrhea and grade 2 abdominal pain.	Linn et al., 2019
VSL#3	Patients were assigned to either the high-potency probiotic preparation VSL#3 or placebo	Probiotic treatment reduces grade 3-4 diarrhea and reduces the number of bowel movements.	Delia et al., 2007
*Lactobacillus acidophilus* LAC-361 and *Bifidobacterium longum* BB-536	Patients were randomized between a placebo and either of two regiments of double strain Bifilact(^®^) probiotics.	Probiotics reduce radiation-induced grade 2-3-4 diarrhea.	Demers et al., 2014
*Lactobacillus casei* DN-114 001	Patients were randomly assigned to a probiotic drink or placebo.	Probiotic intervention had a significant effect on stool consistency.	Giralt et al., 2008

### Application of probiotics in HPV therapeutic vaccine

HPV preventive vaccine can effectively prevent high-risk HPV infection, but cannot improve the treatment effect of cervical cancer. Therefore, researchers are currently focusing on developing therapeutic vaccines (Werner et al., 2012). HPV E6 and E7 oncoproteins are required for maintenance of the tumor phenotype and contribute to the progression of CIN2-3 to cervical cancer. HPV E6 and E7 are considered potential target antigens for therapeutic vaccines. HPV therapeutic vaccines can be divided into protein and peptide vaccines, DNA vaccines and bacterial vector vaccines (Taghinezhad et al., 2021). Among them, the vaccine based on bacteria is widely used. Many preclinical trials have proved that transgenic *Lactobacillus* is relatively safe, has the potential to deliver recombinant antigens, and can induce humoral and cellular immunity in the host and further kill HPV virus (Das et al., 2022). Komatsu et al. (Komatsu et al., 2018) developed a *Lactobacillus casei* (IGMKK16E7) therapeutic HPV vaccine with E7 endogenous expression. It was found that the expression level of E7 molecule was related to the induction efficiency of E7-specific mucosal immune response. Lee et al. (Lee et al) treated mice orally with the HPV16 E6 protein expressed on *Lactobacillus casei* and found that the vaccine could induce the production of E6-specific serum IgG and IgA. (Park et al. (2019) investigated an oral drug (BLS-M07) expressing the HPV 16 E7 antigen on the surface of *Lactobacillus casei* to evaluate its efficacy in CIN3 patients. The results demonstrated that oral administration of BLS-M07 increased the production of serum HPV16E7 specific antibodies. At present, therapeutic vaccine has no clinical application and is still in the stage of clinical trial. Many studies have proved that it is feasible to develop HPV therapeutic vaccine by using engineering bacteria represented by *Lactobacillus*. Future research seems to focus more on the use of such bacteria.

## Effects of probiotics on BV

Bacterial vaginosis (BV) is a mixed infectious diseases caused by the imbalance of normal flora in the vagina, which is characterized by the decrease of *Lactobacillus* and the increase of anaerobic bacteria, especially *Gardnerella* and *Prevotella* (Onderdonk et al., 2016; Bagnall and Rizzolo, 2017). The microbial community structure of BV is basically consistent with CST IV (Coleman and Gaydos, 2018). The traditional treatment method is to use metronidazole and other antibiotics. In fact, the recurrence rate of BV after oral metronidazole treatment is very high, and the systemic use of antibiotics has great side effects (Muzny et al., 2020). In this case, new treatment strategies help to improve treatment outcomes. The use of probiotics can improve vaginal flora, increase beneficial bacteria, reduce the number of harmful bacteria, and further maintain the stability of vaginal flora environment (Ling et al., 2013). Nowadays, there is increasing evidence that probiotics are effective in the treatment of BV. In a meta-analysis of 30 studies (Jeng et al., 2020), BV patients were followed up after treatment and found that probiotic intervention reduced the recurrence rate of vaginitis (OR = 0.27, 95% CI: 0.18-0.41, P<0.001), Improve the cure rate of vaginitis (OR = 2.28, 95% CI: 1.20-4.32, P = 0.011). (Selis et al. (2021) proved through *in vitro* experiments that *Lactobacillus plantarum* Lp62 and its supernatant could significantly inhibit the growth of *Gardnerella*. In another meta-analysis of 18 studies (Liu and Yi, 2022) with 3-month follow-up, the combination of antibiotics and probiotics was found to significantly reduce the recurrence rate of BV compared with antibiotics alone. Inflammation is considered to be a predisposing factor for tumorigenesis and development. Experimental studies in humans and animals support the correlation between chronic inflammation and cancer. Chronic inflammation will increase the gene mutation rate, lead to cancer and promote tumor metastasis. Probiotics combined with antibiotics play an important role in the treatment of inflammation. As shown in **Table 3**, we summarize the clinical research on probiotic treatment of BV in recent years.

**Table 3:** Summarized the clinical effect of probiotics in the treatment of BV.

Probiotics	Methods	Results	Ref.
*Lactobacillus acidophilus* GLA-14 and *Lactobacillus rhamnosus* HN001	Patients received metronidazole (500 mg, bid) for 7 days and were randomly assigned to concurrently receive probiotics plus lactoferrin or placebo (n=48).	Probiotic mixture combined with lactoferrin improved symptoms (vaginal discharge and itching), Nugent scores, and recurrence rates.	Russo et al., 2019
*Lactobacillus rhamnosus* GR-1 and *Lactobacillus reuteri* RC-14	Patients received oral metronidazole for 7 days, and probiotics and placebo for 30 days (n=125).	Combined use of probiotics and antibiotics improves BV cure rates.	Anukam et al., 2006
*Lactobacillus crispatus* LMG S-29995, *Lactobacillus brevis*, and *Lactobacillus acidophilus*	After completing metronidazole treatment, patients received probiotics and placebo, respectively (n=166).	Oral probiotics reduce the rate of BV recurrence and prolong the time to disease recurrence.	Reznichenko et al., 2020
*Lactobacillus rhamnosus* BMX 54	After completing metronidazole treatment, patients received placebo and vaginal tablets containing probiotics, respectively (n=250).	Patients treated with probiotics had reduced BV recurrence rate and vaginal pH.	Recine et al., 2016
*Lactobacillus crispatus* LbV 88, *Lactobacillus gasseri* LbV 150N, *Lactobacillus jensenii* LbV 116 and *Lactobacillus rhamnosus* LbV96	After completing metronidazole treatment, patients received placebo and yogurt with probiotics, respectively (n=36).	Yogurt with probiotics increases BV recovery rates and improves vaginal microbes.	Laue et al., 2018
*L. brevis* CD2, *L. salivarius* subsp. *salicinius*, *L. plantarum*	Patients were randomized to receive probiotic vaginal tablets and vaginal pH tablets (n=64).	Probiotics improve BV cure rates and reduce vaginal cytokines IL-1β and IL-6.	Hemalatha et al., 2012
*Lactobacillus rhamnosus* GR-1 and *L. reuteri* RC-14	Patients were treated with probiotics for 6 months and metronidazole for 10 days (n=65).	Supplementation with probiotics did not improve BV cure rates, but improved vaginal flora composition.	Hummelen et al., 2010
*L. brevis* (CD2), *L. salivarius* subsp.*salicinius* (FV2), and *L. plantarum* (FV9)	Patients received probiotic-containing vaginal tablets or placebo for 7 days (n=39).	Supplementation with probiotics can increase the cure rate of BV and improve the vaginal environment.	Mastromarino et al., 2009

## Effect of probiotics on GSM

GSM was previously known as vulvovaginal atrophy or atrophic vaginitis (Caretto et al., 2017; Donders et al., 2019). When women reach perimenopause, ovarian function declines, resulting in lower estrogen levels. More than 50% of postmenopausal women will have a series of annoying symptoms, including vaginal dryness, pruritus, difficulty in sexual intercourse, urgency and increased frequency of urination, and urinary tract infection (Yoo et al., 2022). Especially after menopause, the decrease of *Lactobacillus* and the increase of other anaerobic bacteria (*Gardnerella* and *Prevotella*) make cervical cells prone to canceration. Currently approved treatment options for GSM include estrogen therapy and non estrogen therapy (Gambrell, 1986). Recent studies have found that probiotics combined with estrogen can alleviate the related symptoms caused by vulvovaginal atrophy. Petricevic and others (Petricevic et al., 2008) found in a randomized controlled study that oral probiotics (*Lactobacillus rhamnosus* Gr-1 and *Lactobacillus reuteri* RC-14) in postmenopausal women could significantly reduce Nugent score and improve GSM symptoms (*P* = 0.0001). In a randomized clinical trial (Ribeiro et al., 2018), the effect of estrogen with or without probiotics on GSM was investigated. Compared with estrogen alone, estrogen combined with probiotics significantly improved GSM symptoms, mainly vaginal dryness and dyspareunia, and increased vaginal health scores. Lim (Lim et al., 2021) also found that the intestinal microbial composition changed after ovariectomy. After supplementing the new intestinal *Lactobacillus* strain to ovariectomized rats, it can significantly reduce the climacteric symptoms, and promote the integrity of the intestinal barrier by increasing the mRNA level of tight junction related markers. In addition to local use of estrogen, oral or vaginal use of probiotics in postmenopausal women is also very effective in reducing menopausal symptoms caused by GSM. This provides a new choice for improving the quality of life of postmenopausal women.

## Vaginal microbial transplantation

Fecal microbiota transplantation (FMT) has attracted more and more attention in the treatment of other diseases such as digestive system diseases, and has also achieved remarkable results. There has also been a growing interest in vaginal microbial transplantation (VMT) in recent years (Korpela et al., 2020; Wang et al., 2021). One study (Chen et al., 2021) investigated the effect of VMT on vaginal dysbiosis by establishing a vaginal dysbiosis model. The results showed that VMT significantly reduced bacterial-induced inflammation, as well as the enrichment of pro-inflammatory cytokines, and restored normal vaginal microbiota. Lev-Sagie et al. (Lev-Sagie et al., 2019) were the first to report the use of VMT from healthy donors as an alternative to the treatment of patients with BV. After 5-21 months of follow-up, patients who received VMT showed improved vaginal fluid appearance and reconstituted vaginal microbiota dominated by *Lactobacilli.* Huang et al. (Huang et al., 2021) transplanted the feces of female mice with intact and prolific ovaries into the feces of ovariectomized mice and found that vaginal epithelial atrophy was significantly reduced and intestinal flora was significantly altered. In contrast to the studies described above, in a randomized controlled trial (Wilson et al., 2021) the gut microbiota of infants born by caesarean section was assessed at 2 hours, 1 month and 3 months after oral administration of maternal vaginal microbes. The results showed that oral administration of maternal vaginal secretions did not alter the gut microbiota composition of early infants compared with oral placebo infants. The findings of this study question the value of vaginal vaccination.

## Conclusion

There is an association between a highly diverse vaginal microbiota and female reproductive tract health. Probiotics play an important role in maintaining the health of the female reproductive tract, alleviating gynecological diseases, and enhancing the local immunity of the vagina. The use of probiotics or VMT intervention has a certain effect on preventing the progression of CIN, treating BV, and relieving symptoms related to senile vaginitis. The development of 16SrRNA sequencing technology can help to identify microbial markers and carry out personalized prevention and treatment of diseases. At present, the mechanism of action of probiotics in cervical cancer is not fully understood. In the future, it is necessary to conduct larger-scale clinical studies and longitudinal tracking. Combination immunotherapy and multi-omics analysis are also necessary to fully understand the relationship between host, vaginal microbes and disease.

## Author contributions

ZM organized the literature. ZM and DL co-authored the article.

## Conflict of interest

The authors declare that the research was conducted in the absence of any commercial or financial relationships that could be construed as a potential conflict of interest.

## Publisher’s note

All claims expressed in this article are solely those of the authors and do not necessarily represent those of their affiliated organizations, or those of the publisher, the editors and the reviewers. Any product that may be evaluated in this article, or claim that may be made by its manufacturer, is not guaranteed or endorsed by the publisher.

## References

[B1] Abdolalipour E. Mahooti M. Salehzadeh A. Torabi A. Mohebbi S. R. Gorji A.. (2020). Evaluation of the antitumor immune responses of probiotic bifidobacterium bifidum in human papillomavirus-induced tumor model. Microb. Pathog. 145, 104207. doi: 10.1016/j.micpath.2020.104207 32325236

[B2] Anderson D. J. Marathe J. Pudney J. (2014). The structure of the human vaginal stratum corneum and its role in immune defense. Am. J. Reprod. Immunol. 71 (6), 618–623. doi: 10.1111/aji.12230 24661416 PMC4024347

[B3] Anukam K. Osazuwa E. Ahonkhai I. Ngwu M. Osemene G. Bruce A. W.. (2006). Augmentation of antimicrobial metronidazole therapy of bacterial vaginosis with oral probiotic lactobacillus rhamnosus GR-1 and lactobacillus reuteri RC-14: randomized, double-blind, placebo controlled trial. Microbes Infect. 8 (6), 1450–1454. doi: 10.1016/j.micinf.2006.01.003 16697231

[B4] Athanasiou S. Pitsouni E. Antonopoulou S. Zacharakis D. Salvatore S. Falagas M. E.. (2016). The effect of microablative fractional CO2 laser on vaginal flora of postmenopausal women. Climacteric 19 (5), 512–518. doi: 10.1080/13697137.2016.1212006 27558459

[B5] Bagnall P. Rizzolo D. (2017). Bacterial vaginosis: A practical review. Jaapa 30 (12), 15–21. doi: 10.1097/01.JAA.0000526770.60197.fa 29135564

[B6] Borgogna J. C. Shardell M. D. Santori E. K. Nelson T. M. Rath J. M. Glover E. D.. (2020). The vaginal metabolome and microbiota of cervical HPV-positive and HPV-negative women: a cross-sectional analysis. Bjog 127 (2), 182–192. doi: 10.1111/1471-0528.15981 31749298 PMC6982399

[B7] Caretto M. Giannini A. Russo E. Simoncini T. (2017). Preventing urinary tract infections after menopause without antibiotics. Maturitas 99, 43–46. doi: 10.1016/j.maturitas.2017.02.004 28364867

[B8] Cha M. K. Lee D. K. An H. M. Lee S. W. Shin S. H. Kwon J. H.. (2012). Antiviral activity of bifidobacterium adolescentis SPM1005-a on human papillomavirus type 16. BMC Med. 10, 72. doi: 10.1186/1741-7015-10-72 22788922 PMC3409845

[B9] Champer M. Wong A. M. Champer J. Brito I. L. Messer P. W. Hou J. Y.. (2018). The role of the vaginal microbiome in gynaecological cancer. Bjog 125 (3), 309–315. doi: 10.1111/1471-0528.14631 28278350

[B10] Chao X. P. Sun T. T. Wang S. Fan Q. B. Shi H. H. Zhu L.. (2019). Correlation between the diversity of vaginal microbiota and the risk of high-risk human papillomavirus infection. Int. J. Gynecol Cancer 29 (1), 28–34. doi: 10.1136/ijgc-2018-000032 30640680

[B11] Chen T. Xia C. Hu H. Wang H. Tan B. Tian P.. (2021). Dysbiosis of the rat vagina is efficiently rescued by vaginal microbiota transplantation or probiotic combination. Int. J. Antimicrob. Agents 57 (3), 106277. doi: 10.1016/j.ijantimicag.2021.106277 33434677

[B12] Chitapanarux I. Chitapanarux T. Traisathit P. Kudumpee S. Tharavichitkul E. Lorvidhaya V. (2010). Randomized controlled trial of live lactobacillus acidophilus plus bifidobacterium bifidum in prophylaxis of diarrhea during radiotherapy in cervical cancer patients. Radiat. Oncol. 5, 31. doi: 10.1186/1748-717x-5-31 20444243 PMC2874795

[B13] Coleman J. S. Gaydos C. A. (2018). Molecular diagnosis of bacterial vaginosis: an update. J. Clin. Microbiol. 56 (9), e00342–18. doi: 10.1128/jcm.00342-18 PMC6113459 29769280

[B14] Curty G. de Carvalho P. S. Soares M. A. (2019). The role of the cervicovaginal microbiome on the genesis and as a biomarker of premalignant cervical intraepithelial neoplasia and invasive cervical cancer. Int. J. Mol. Sci. 21 (1), 222. doi: 10.3390/ijms21010222 PMC6981542 31905652

[B15] Das S. Bhattacharjee M. J. Mukherjee A. K. Khan M. R. (2022). Recent advances in understanding of multifaceted changes in the vaginal microenvironment: implications in vaginal health and therapeutics. Crit. Rev. Microbiol. 21, 1–27. doi: 10.1080/1040841x.2022.2049696 35312419

[B16] Delia P. Sansotta G. Donato V. Frosina P. Messina G. De Renzis C.. (2007). Use of probiotics for prevention of radiation-induced diarrhea. World J. Gastroenterol. 13 (6), 912–915. doi: 10.3748/wjg.v13.i6.912 17352022 PMC4065928

[B17] Demers M. Dagnault A. Desjardins J. (2014). A randomized double-blind controlled trial: impact of probiotics on diarrhea in patients treated with pelvic radiation. Clin. Nutr. 33 (5), 761–767. doi: 10.1016/j.clnu.2013.10.015 24200199

[B18] DI Pierro F. Criscuolo A. A. Dei Giudici A. Senatori R. Sesti F. Ciotti M.. (2021). Oral administration of lactobacillus crispatus M247 to papillomavirus-infected women: results of a preliminary, uncontrolled, open trial. Minerva Obstet Gynecol 73 (5), 621–631. doi: 10.23736/s2724-606x.21.04752-7 33876901

[B19] Donders G. G. G. Ruban K. Bellen G. Grinceviciene S. (2019). Pharmacotherapy for the treatment of vaginal atrophy. Expert Opin. Pharmacother. 20 (7), 821–835. doi: 10.1080/14656566.2019.1574752 30897020

[B20] Gambrell R. D. Jr. (1986). The menopause. Invest. Radiol. 21 (4), 369–378. doi: 10.1097/00004424-198604000-00017 3516923

[B21] Gao W. Weng J. Gao Y. Chen X. (2013). Comparison of the vaginal microbiota diversity of women with and without human papillomavirus infection: a cross-sectional study. BMC Infect. Dis. 13, 271. doi: 10.1186/1471-2334-13-271 23758857 PMC3684509

[B22] Giralt J. Regadera J. P. Verges R. Romero J. de la Fuente I. Biete A.. (2008). Effects of probiotic lactobacillus casei DN-114 001 in prevention of radiation-induced diarrhea: results from multicenter, randomized, placebo-controlled nutritional trial. Int. J. Radiat. Oncol. Biol. Phys. 71 (4), 1213–1219. doi: 10.1016/j.ijrobp.2007.11.009 18243569

[B23] Han Y. Ren Q. L. (2021). Does probiotics work for bacterial vaginosis and vulvovaginal candidiasis. Curr. Opin. Pharmacol. 61, 83–90. doi: 10.1016/j.coph.2021.09.004 34649216

[B24] Hemalatha R. Mastromarino P. Ramalaxmi B. A. Balakrishna N. V. Sesikeran B. (2012). Effectiveness of vaginal tablets containing lactobacilli versus pH tablets on vaginal health and inflammatory cytokines: a randomized, double-blind study. Eur. J. Clin. Microbiol. Infect. Dis. 31 (11), 3097–3105. doi: 10.1007/s10096-012-1671-1 22777592

[B25] Hombrink J. Fröhlich D. Glatzel M. Krauss A. Thiel H. J. Meier J.. (2000). Prevention of radiation-induced diarrhea by smectite. results of a double-blind randomized, placebo-controlled multicenter study. Strahlenther Onkol 176 (4), 173–179. doi: 10.1007/s000660050053 10812390

[B26] Huang J. Shan W. Li F. Wang Z. Cheng J. Lu F.. (2021). Fecal microbiota transplantation mitigates vaginal atrophy in ovariectomized mice. Aging (Albany NY) 13 (5), 7589–7607. doi: 10.18632/aging.202627 33658399 PMC7993734

[B27] Hummelen R. Changalucha J. Butamanya N. L. Cook A. Habbema J. D. Reid G. (2010). Lactobacillus rhamnosus GR-1 and l. reuteri RC-14 to prevent or cure bacterial vaginosis among women with HIV. Int. J. Gynaecol Obstet 111 (3), 245–248. doi: 10.1016/j.ijgo.2010.07.008 20801446

[B28] Ilhan Z. E. Łaniewski P. Thomas N. Roe D. J. Chase D. M. Herbst-Kralovetz M. M. (2019). Deciphering the complex interplay between microbiota, HPV, inflammation and cancer through cervicovaginal metabolic profiling. EBioMedicine 44, 675–690. doi: 10.1016/j.ebiom.2019.04.028 31027917 PMC6604110

[B29] Jahanshahi M. Maleki Dana P. Badehnoosh B. Asemi Z. Hallajzadeh J. Mansournia M. A.. (2020). Anti-tumor activities of probiotics in cervical cancer. J. Ovarian Res. 13 (1), 68. doi: 10.1186/s13048-020-00668-x 32527332 PMC7291573

[B30] Jang S. E. Jeong J. J. Choi S. Y. Kim H. Han M. J. Kim D. H. (2017). Lactobacillus rhamnosus HN001 and lactobacillus acidophilus la-14 attenuate gardnerella vaginalis-infected bacterial vaginosis in mice. Nutrients 9 (6), 531. doi: 10.3390/nu9060531 PMC5490510 28545241

[B31] Jeng H. S. Yan T. R. Chen J. Y. (2020). Treating vaginitis with probiotics in non-pregnant females: A systematic review and meta-analysis. Exp. Ther. Med. 20 (4), 3749–3765. doi: 10.3892/etm.2020.9090 32855726 PMC7444381

[B32] Kandati K. Belagal P. Nannepaga J. S. Viswanath B. (2022). Role of probiotics in the management of cervical cancer: An update. Clin. Nutr. ESPEN 48, 5–16. doi: 10.1016/j.clnesp.2022.02.017 35331533

[B33] Komatsu A. Igimi S. Kawana K. (2018). Optimization of human papillomavirus (HPV) type 16 E7-expressing lactobacillus-based vaccine for induction of mucosal E7-specific IFNγ-producing cells. Vaccine 36 (24), 3423–3426. doi: 10.1016/j.vaccine.2018.05.009 29735324

[B34] Korpela K. Helve O. Kolho K. L. Saisto T. Skogberg K. Dikareva E.. (2020). Maternal fecal microbiota transplantation in cesarean-born infants rapidly restores normal gut microbial development: A proof-of-Concept study. Cell 183 (2), 324–334.e325. doi: 10.1016/j.cell.2020.08.047 33007265

[B35] Kovachev S. M. (2020). Cervical cancer and vaginal microbiota changes. Arch. Microbiol. 202 (2), 323–327. doi: 10.1007/s00203-019-01747-4 31659380

[B36] Langner C. A. Ortiz A. M. Flynn J. K. Kendall H. Lagenaur L. A. Brenchley J. M. (2021). The vaginal microbiome of nonhuman primates can be only transiently altered to become lactobacillus dominant without reducing inflammation. Microbiol. Spectr. 9 (3), e0107421. doi: 10.1128/Spectrum.01074-21 34756073 PMC8579922

[B37] Łaniewski P. Ilhan Z. E. Herbst-Kralovetz M. M. (2020). The microbiome and gynaecological cancer development, prevention and therapy. Nat. Rev. Urol 17 (4), 232–250. doi: 10.1038/s41585-020-0286-z 32071434 PMC9977514

[B38] Laue C. Papazova E. Liesegang A. Pannenbeckers A. Arendarski P. Linnerth B.. (2018). Effect of a yoghurt drink containing lactobacillus strains on bacterial vaginosis in women - a double-blind, randomised, controlled clinical pilot trial. Benef Microbes 9 (1), 35–50. doi: 10.3920/bm2017.0018 29065710

[B39] Lev-Sagie A. Goldman-Wohl D. Cohen Y. Dori-Bachash M. Leshem A. Mor U.. (2019). Vaginal microbiome transplantation in women with intractable bacterial vaginosis. Nat. Med. 25 (10), 1500–1504. doi: 10.1038/s41591-019-0600-6 31591599

[B40] Lim E. Y. Song E. J. Kim J. G. Jung S. Y. Lee S. Y. Shin H. S.. (2021). Lactobacillus intestinalis YT2 restores the gut microbiota and improves menopausal symptoms in ovariectomized rats. Benef Microbes 12 (5), 503–516. doi: 10.3920/bm2020.0217 34463192

[B41] Ling Z. Liu X. Chen W. Luo Y. Yuan L. Xia Y.. (2013). The restoration of the vaginal microbiota after treatment for bacterial vaginosis with metronidazole or probiotics. Microb. Ecol. 65 (3), 773–780. doi: 10.1007/s00248-012-0154-3 23250116

[B42] Linn Y. H. Thu K. K. Win N. H. H. (2019). Effect of probiotics for the prevention of acute radiation-induced diarrhoea among cervical cancer patients: a randomized double-blind placebo-controlled study. Probiotics Antimicrob. Proteins 11 (2), 638–647. doi: 10.1007/s12602-018-9408-9 29550911

[B43] Liu J. Luo M. Zhang Y. Cao G. Wang S. (2020). Association of high-risk human papillomavirus infection duration and cervical lesions with vaginal microbiota composition. Ann. Transl. Med. 8 (18), 1161. doi: 10.21037/atm-20-5832 33241010 PMC7576078

[B44] Liu H. F. Yi N. (2022). A systematic review and meta-analysis on the efficacy of probiotics for bacterial vaginosis. Eur. Rev. Med. Pharmacol. Sci. 26 (1), 90–98. doi: 10.26355/eurrev_202201_27752 35049024

[B45] Li X. Wang H. Du X. Yu W. Jiang J. Geng Y.. (2017). Lactobacilli inhibit cervical cancer cell migration *in vitro* and reduce tumor burden *in vivo* through upregulation of e-cadherin. Oncol. Rep. 38 (3), 1561–1568. doi: 10.3892/or.2017.5791 28713905

[B46] Li Y. Yu T. Yan H. Li D. Yu T. Yuan T.. (2020). Vaginal microbiota and HPV infection: Novel mechanistic insights and therapeutic strategies. Infect. Drug Resist. 13, 1213–1220. doi: 10.2147/idr.s210615 32431522 PMC7198448

[B47] Mastromarino P. Macchia S. Meggiorini L. Trinchieri V. Mosca L. Perluigi M.. (2009). Effectiveness of lactobacillus-containing vaginal tablets in the treatment of symptomatic bacterial vaginosis. Clin. Microbiol. Infect. 15 (1), 67–74. doi: 10.1111/j.1469-0691.2008.02112.x 19046169

[B48] Medina-Contreras O. Luvián-Morales J. Valdez-Palomares F. Flores-Cisneros L. Sánchez-López M. S. Soto-Lugo J. H.. (2020). IMMUNONUTRITION IN CERVICAL CANCER: IMMUNE RESPONSE MODULATION BY DIET. Rev. Invest. Clin. 72 (4), 219–230. doi: 10.24875/ric.20000062 33064687

[B49] Mitra A. MacIntyre D. A. Lee Y. S. Smith A. Marchesi J. R. Lehne B.. (2015). Cervical intraepithelial neoplasia disease progression is associated with increased vaginal microbiome diversity. Sci. Rep. 5, 16865. doi: 10.1038/srep16865 26574055 PMC4648063

[B50] Mitra A. MacIntyre D. A. Ntritsos G. Smith A. Tsilidis K. K. Marchesi J. R.. (2020). The vaginal microbiota associates with the regression of untreated cervical intraepithelial neoplasia 2 lesions. Nat. Commun. 11 (1), 1999. doi: 10.1038/s41467-020-15856-y 32332850 PMC7181700

[B51] Motevaseli E. Azam R. Akrami S. M. Mazlomy M. Saffari M. Modarressi M. H.. (2016). The effect of lactobacillus crispatus and lactobacillus rhamnosusCulture supernatants on expression of autophagy genes and HPV E6 and E7 oncogenes in the HeLa cell line. Cell J. 17 (4), 601–607. doi: 10.22074/cellj.2016.3833 26862519 PMC4746410

[B52] Motevaseli E. Shirzad M. Akrami S. M. Mousavi A. S. Mirsalehian A. Modarressi M. H. (2013). Normal and tumour cervical cells respond differently to vaginal lactobacilli, independent of pH and lactate. J. Med. Microbiol. 62 (Pt 7), 1065–1072. doi: 10.1099/jmm.0.057521-0 23618799

[B53] Muzny C. A. Łaniewski P. Schwebke J. R. Herbst-Kralovetz M. M. (2020). Host-vaginal microbiota interactions in the pathogenesis of bacterial vaginosis. Curr. Opin. Infect. Dis. 33 (1), 59–65. doi: 10.1097/qco.0000000000000620 31789672 PMC7265982

[B54] Nami Y. Abdullah N. Haghshenas B. Radiah D. Rosli R. Khosroushahi A. Y. (2014). Assessment of probiotic potential and anticancer activity of newly isolated vaginal bacterium lactobacillus plantarum 5BL. Microbiol. Immunol. 58 (9), 492–502. doi: 10.1111/1348-0421.12175 25039934

[B55] Negi D. Singh A. Joshi N. Mishra N. (2020). Cisplatin and probiotic biomass loaded pessaries for the management of cervical cancer. Anticancer Agents Med. Chem. 20 (5), 589–598. doi: 10.2174/1871520619666191211110640 31823703

[B56] Niu X. X. Li T. Zhang X. Wang S. X. Liu Z. H. (2017). Lactobacillus crispatus modulates vaginal epithelial cell innate response to candida albicans. Chin. Med. J. (Engl) 130 (3), 273–279. doi: 10.4103/0366-6999.198927 28139509 PMC5308008

[B57] Nouri Z. Karami F. Neyazi N. Modarressi M. H. Karimi R. Khorramizadeh M. R.. (2016). Dual anti-metastatic and anti-proliferative activity assessment of two probiotics on HeLa and HT-29 cell lines. Cell J. 18 (2), 127–134. doi: 10.22074/cellj.2016.4307 27551673 PMC4992182

[B58] Okawa T. Niibe H. Arai T. Sekiba K. Noda K. Takeuchi S.. (1993). Effect of LC9018 combined with radiation therapy on carcinoma of the uterine cervix. a phase III, multicenter, randomized, controlled study. Cancer 72 (6), 1949–1954. doi: 10.1002/1097-0142(19930915)72:6<1949::aid-cncr2820720626>3.0.co;2-w 8364872

[B59] Onderdonk A. B. Delaney M. L. Fichorova R. N. (2016). The human microbiome during bacterial vaginosis. Clin. Microbiol. Rev. 29 (2), 223–238. doi: 10.1128/cmr.00075-15 26864580 PMC4786887

[B60] Park Y. C. Ouh Y. T. Sung M. H. Park H. G. Kim T. J. Cho C. H.. (2019). A phase 1/2a, dose-escalation, safety and preliminary efficacy study of oral therapeutic vaccine in subjects with cervical intraepithelial neoplasia 3. J. Gynecol Oncol. 30 (6), e88. doi: 10.3802/jgo.2019.30.e88 31576684 PMC6779607

[B61] Petricevic L. Unger F. M. Viernstein H. Kiss H. (2008). Randomized, double-blind, placebo-controlled study of oral lactobacilli to improve the vaginal flora of postmenopausal women. Eur. J. Obstet Gynecol Reprod. Biol. 141 (1), 54–57. doi: 10.1016/j.ejogrb.2008.06.003 18701205

[B62] Piccioni A. Franza L. Vaccaro V. Saviano A. Zanza C. Candelli M.. (2021). Microbiota and probiotics: The role of limosilactobacillus reuteri in diverticulitis. Medicina (Kaunas) 57 (8), 802. doi: 10.3390/medicina57080802 34441008 PMC8398895

[B63] Pourmollaei S. Barzegari A. Farshbaf-Khalili A. Nouri M. Fattahi A. Shahnazi M.. (2020). Anticancer effect of bacteria on cervical cancer: Molecular aspects and therapeutic implications. Life Sci. 246, 117413. doi: 10.1016/j.lfs.2020.117413 32035929

[B64] Pramanick R. Mayadeo N. Warke H. Begum S. Aich P. Aranha C. (2019). Vaginal microbiota of asymptomatic bacterial vaginosis and vulvovaginal candidiasis: Are they different from normal microbiota? Microb. Pathog. 134, 103599. doi: 10.1016/j.micpath.2019.103599 31212037

[B65] Qiu G. Yu Y. Wang Y. Wang X. (2019). The significance of probiotics in preventing radiotherapy-induced diarrhea in patients with cervical cancer: A systematic review and meta-analysis. Int. J. Surg. 65, 61–69. doi: 10.1016/j.ijsu.2019.03.015 30928672

[B66] Recine N. Palma E. Domenici L. Giorgini M. Imperiale L. Sassu C.. (2016). Restoring vaginal microbiota: biological control of bacterial vaginosis. a prospective case-control study using lactobacillus rhamnosus BMX 54 as adjuvant treatment against bacterial vaginosis. Arch. Gynecol Obstet 293 (1), 101–107. doi: 10.1007/s00404-015-3810-2 26142892

[B67] Reznichenko H. Henyk N. Maliuk V. Khyzhnyak T. Tynna Y. Filipiuk I.. (2020). Oral intake of lactobacilli can be helpful in symptomatic bacterial vaginosis: A randomized clinical study. J. Low Genit Tract Dis. 24 (3), 284–289. doi: 10.1097/lgt.0000000000000518 32091443

[B68] Riaz Rajoka M. S. Zhao H. Lu Y. Lian Z. Li N. Hussain N.. (2018). Anticancer potential against cervix cancer (HeLa) cell line of probiotic lactobacillus casei and lactobacillus paracasei strains isolated from human breast milk. Food Funct. 9 (5), 2705–2715. doi: 10.1039/c8fo00547h 29762617

[B69] Ribeiro A. E. Monteiro N. E. S. Moraes A. V. G. Costa-Paiva L. H. Pedro A. O. (2018). Can the use of probiotics in association with isoflavone improve the symptoms of genitourinary syndrome of menopause? results from a randomized controlled trial. Menopause 26 (6), 643–652. doi: 10.1097/gme.0000000000001279 30531444

[B70] Russo R. Karadja E. De Seta F. (2019). Evidence-based mixture containing lactobacillus strains and lactoferrin to prevent recurrent bacterial vaginosis: a double blind, placebo controlled, randomised clinical trial. Benef Microbes 10 (1), 19–26. doi: 10.3920/bm2018.0075 30525953

[B71] Saraf V. S. Sheikh S. A. Ahmad A. Gillevet P. M. Bokhari H. Javed S. (2021). Vaginal microbiome: normalcy vs dysbiosis. Arch. Microbiol. 203 (7), 3793–3802. doi: 10.1007/s00203-021-02414-3 34120200

[B72] Selis N. N. Oliveira H. B. M. Souza C. L. S. Almeida J. B. Andrade Y. Silva L. S. C.. (2021). Lactobacillus plantarum Lp62 exerts probiotic effects against gardnerella vaginalis ATCC 49154 in bacterial vaginosis. Lett. Appl. Microbiol. 73 (5), 579–589. doi: 10.1111/lam.13547 34338346

[B73] Sungur T. Aslim B. Karaaslan C. Aktas B. (2017). Impact of exopolysaccharides (EPSs) of lactobacillus gasseri strains isolated from human vagina on cervical tumor cells (HeLa). Anaerobe 47, 137–144. doi: 10.1016/j.anaerobe.2017.05.013 28554813

[B74] Taghinezhad S. S. Keyvani H. Bermúdez-Humarán L. G. Donders G. G. G. Fu X. Mohseni A. H. (2021). Twenty years of research on HPV vaccines based on genetically modified lactic acid bacteria: an overview on the gut-vagina axis. Cell Mol. Life Sci. 78 (4), 1191–1206. doi: 10.1007/s00018-020-03652-2 32979054 PMC7519697

[B75] Wang J. Li Z. Ma X. Du L. Jia Z. Cui X.. (2021). Translocation of vaginal microbiota is involved in impairment and protection of uterine health. Nat. Commun. 12 (1), 4191. doi: 10.1038/s41467-021-24516-8 34234149 PMC8263591

[B76] Wang H. Ma Y. Li R. Chen X. Wan L. Zhao W. (2019). Associations of cervicovaginal lactobacilli with high-risk human papillomavirus infection, cervical intraepithelial neoplasia, and cancer: A systematic review and meta-analysis. J. Infect. Dis. 220 (8), 1243–1254. doi: 10.1093/infdis/jiz325 31242505

[B77] Wang K. D. Xu D. J. Wang B. Y. Yan D. H. Lv Z. Su J. R. (2018). Inhibitory effect of vaginal lactobacillus supernatants on cervical cancer cells. Probiotics Antimicrob. Proteins 10 (2), 236–242. doi: 10.1007/s12602-017-9339-x 29071554

[B78] Wei Z. T. Chen H. L. Wang C. F. Yang G. L. Han S. M. Zhang S. L. (2020). Depiction of vaginal microbiota in women with high-risk human papillomavirus infection. Front. Public Health 8. doi: 10.3389/fpubh.2020.587298 PMC7820762 33490017

[B79] Werner J. Decarlo C. A. Escott N. Zehbe I. Ulanova M. (2012). Expression of integrins and toll-like receptors in cervical cancer: effect of infectious agents. Innate Immun. 18 (1), 55–69. doi: 10.1177/1753425910392934 21239458

[B80] Wilson B. C. Butler É M. Grigg C. P. Derraik J. G. B. Chiavaroli V. Walker N.. (2021). Oral administration of maternal vaginal microbes at birth to restore gut microbiome development in infants born by caesarean section: A pilot randomised placebo-controlled trial. EBioMedicine 69, 103443. doi: 10.1016/j.ebiom.2021.103443 34186487 PMC8254083

[B81] Xie Y. Feng Y. Li W. Zhan F. Huang G. Hu H.. (2020). Revealing the disturbed vaginal micobiota caused by cervical cancer using high-throughput sequencing technology. Front. Cell Infect. Microbiol. 10. doi: 10.3389/fcimb.2020.538336 PMC7750457 33365275

[B82] Yoo S. H. Kim K. R. Park N. J. (2022). Transitional cell metaplasia of the uterine cervix: A histopathological and immunohistochemical analysis suggesting a possible role of androgenic conversion during urothelial-like differentiation in peri/postmenopausal women. Ann. Diagn. Pathol. 56, 151839. doi: 10.1016/j.anndiagpath.2021.151839 34784541

[B83] Zhang Z. Li T. Zhang D. Zong X. Bai H. Bi H.. (2021). Distinction between vaginal and cervical microbiota in high-risk human papilloma virus-infected women in China. BMC Microbiol. 21 (1), 90. doi: 10.1186/s12866-021-02152-y 33765914 PMC7993496

